# The impact of active components from *Piper sarmentosum* on the growth, intestinal barrier function, and immunity of broiler chickens

**DOI:** 10.5713/ab.24.0736

**Published:** 2025-02-27

**Authors:** Luli Zhou, Guanyu Hou, Hanlin Zhou, Khaled Abouelezz, Yuxiu Ye, Dingfa Wang

**Affiliations:** 1 Tropical Crops Genetic Resources Institute, Chinese Academy of Tropical Agricultural Sciences, Haikou, China; 2 Zhanjiang Experimental Station, Chinese Academy of Tropical Agricultural Sciences, Zhanjiang, China; 3 Department of Poultry Production, Faculty of Agriculture, Assiut University, Assiut, Egypt; 4 Hainan Yitian Biotechnology Co., Ltd., Haikou, China

**Keywords:** Chickens, Growth Performance, Intestinal Barrier and Immune Function, Pellitorine, Vitexin-2-O-Rhamnoside

## Abstract

**Objective:**

Pellitorine (PT) and vitexin-2-O-rhamnoside (VR) are two bioactive compounds found in *Piper sarmentosum* (PS). Their contents are relatively high in the ethanol extract of PS (PSE). However, it remains unknown whether PT and VR are the primary components of PS that exert beneficial effects on gut health. In this study, we aimed to confirm that these two compounds are the primary anti-inflammatory active ingredients in PSE.

**Methods:**

Total of 300 female one-day-old Danzhou chickens were randomly divided into five groups with five replicates each. Chickens were given a basal diet (CON group), a basal diet with added PSE (200 mg/kg), VR (1.321 mg/kg), PT (0.563 mg/kg), and a combination of VR+PT (1.321 mg/kg VR and 0.563 mg/kg PT) until they reached 35 days of age.

**Results:**

The findings reveal that the VR+PT group exhibited increased liver, thymus and spleen indices compared to the CON group, along with elevated mRNA levels of *ZO-1* and *Claudin-1* (p<0.05). In contrast, the VR+PT group exhibited reduced serum levels and the ileum mucosa mRNA expression levels of IL-1β and IL-6 compared to the CON group (p<0.05). Additionally, the chickens in the VR+PT group had a greater final weight and average daily gain than those in the CON and PSE groups, with a significantly lower level of *D*-lactic acid in serum (p<0.05). The serum IgM level increased significantly (p<0.05) in the VR+PT group compared to the PSE group, while the kidney epinephrine level decreased significantly (p<0.05).

**Conclusion:**

The present study provides preliminary evidence that VR and PT are two of the main active compounds in PSE, which can cooperatively improve growth performance, intestinal barrier integrity, and immune function in chickens.

## INTRODUCTION

Due to the escalating issue of drug resistance resulting from the misuse of antibiotics, many countries and regions worldwide have implemented bans or limitations on antibiotic growth promoters in animal feed [1]. Herbal extracts from plants have become increasingly popular as substitutes for antibiotics due to their diverse biological functions, such as anti-inflammatory and immunomodulatory properties, with minimal negative effects [2–4]. Plant extract is known to be a complex mixture that possess vital properties of multi-component and multi-target. Therefore, understanding the key active ingredients and how plant extracts work is essential for enhancing their overall effectiveness, quality, and consistency [5–7].

*Piper sarmentosum* (PS) is a medicinal and food dual-purpose and often utilized in traditional medicine to alleviate symptoms such as cough, pleurisy, toothache, and asthma [8]. Our published studies have demonstrated that, *Piper sarmentosum* extract (PSE) is rich in natural compounds, such as phenolics, alkaloids and amides [9]. A diet supplemented with PSE at 100 to 300 mg/kg exhibits pleiotropic effects in regulating the immune system of chickens, including the amelioration of intestinal barrier dysfunction, as well as anti-inflammatory and antioxidant effects. Among these, a dietary supplementation level of 200 mg/kg PSE resulted in the best anti-inflammatory effect for chickens [10,11]. Further quantitative analysis also found that vitexin 2-*O*-rhamnoside (VR) (6.605 mg/g) and pellitorine (PT) (2.815 mg/g) were the two compounds with the highest abundance among the six compounds (i.e., 8-hydroxyquinoline, norharman, vitexin, VR, genistin, and PT) detected in the PSE [12]. Besides, among the three extracted-fractions of PSE (which were partitioned with petroleum ether, ethyl acetate, and n-butanol, in succession), the n-butanol fraction of PSE (PSE-NB) was found to have the strongest anti-inflammatory activity [9,13]. Notably, our recent *in vitro* studies revealed that the anti-inflammatory effect of VR in combination with PT was superior to that of PSE-NB (unpublished data). From the above, it can be seen that VR and PT might be the primary active components that simultaneously coexist in PSE for immunomodulatory roles. However, the role of VR in combination with PT *in vivo* has not yet been described in the literature and requires further investigation.

Remarkably, it has been demonstrated that PT could across the blood-brain barrier and affect the central nervous system [14], while VR suffers from poor absorption and low bioavailability in the intestine and tends to be distributed in the liver, spleen, and kidney without crossing the blood-brain barrier [15]. As a result, these findings of VR and PT provided useful information for further study of the pharmacodynamics interaction of both, and we hypothesized that microbiota-gut-brain axis may be a potential target through which VR and PT exert their biological activities. Therefore, in this study, we focused on examining how VR and PT, both separately and together, impact the growth performance, intestinal barrier function, and immunity of chickens. The findings from the study may offer empirical support for utilizing PSE as a dietary supplement for poultry.

## MATERIALS AND METHODS

### Animals and experimental design

The Animal Care and Use Committee of the Chinese Academy of Tropical Agricultural Sciences reviewed and approved all experimental protocols (approval number: CATAS-20220301-1). The Breeding and Demonstration Base of the Tropical Crops Genetic Resources Institute, Chinese Academy of Tropical Agricultural Sciences in Danzhou, Hainan, China, supplied Danzhou chickens. PSE was prepared, and component analysis was conducted as previously described [10,12]. In short, dried PS is extracted by ethanol solution, rotary evaporation, and freeze-drying to obtain PSE. VR was acquired from Aladin Biochemical Technology Co. Ltd. In Shanghai, China (purity≥98%), while PT was obtained from Sigma-Aldrich in St Louis, MO, USA (purity≥98%).

A total of 300 1-day-old female Danzhou chickens with an average initial body weight of 26.06±0.61 g, were randomly divided into five groups for a 35-day experiment, with each group having five replicates of 12 birds each: the control group (CON) receiving a basal diet; the PSE group receiving a basal diet with 200 mg/kg PSE; the VR group receiving a basal diet with 1.321 mg/kg VR; the PT group receiving a basal diet with 0.563 mg/kg PT; and the VR+ PT group receiving a basal diet with 1.321 mg/kg VR and 0.563 mg/kg PT. Table 1 displays the basal diet composition and corresponding nutrient levels. The PSE dosage of 200 mg/kg was selected based on our prior research results [10,11], and the experimental doses of VR and PT were equivalent to the content in 200 mg of PSE. In our previous research, we conducted quantitative analysis of VR and PT in PSE [12].

During the 35-day experiment, every bird was provided with unrestricted food and water while being raised in isolator cages in a pathogen-free environment. The room temperature starts at 36°C, decreases by 2 to 3°C per week, and finally stabilizes at 26±2°C, with a relative humidity of 70±10%, and a 12/12 h light/dark cycle.

### Growth performance

The consumption of food and the weight of the body were documented at the start and end of the 35-day trial period. For each replicate, the average daily gain (ADG), average daily feed intake, and feed efficiency (F/G) were determined from day 1 to day 35.

### Sample collection

At the end of the trial, one broiler from each replicate was chosen at random and its weight was measured. Samples of blood were taken from the bird’s jugular vein, then serum was extracted by centrifuging at 4,000×g for 10 minutes and preserved at −80°C. Following the collection of blood samples, the birds were promptly euthanized through cervical dislocation to obtain tissues. The liver, spleen, thymus, and bursa of fabricius were dissected, blotted, and then promptly weighed using a BP221S balance from Sartorius in Gottingen, Germany. The contents of ileum were gathered, placed in sterile tubes, and frozen at −80°C for later examination. Samples of cerebellum, hypothalamus, kidney, and ileal mucosa were collected, quickly frozen in liquid nitrogen, and kept at −80°C for analysis. Sections measuring 2 cm in length were taken from the duodenum, jejunum, and ileum, rinsed with 0.1 M phosphate-buffered saline (pH 7.2), and fixed in 10% formaldehyde-phosphate buffer for subsequent histological analysis.

### Histological examination

Hematoxylin-eosin staining was utilized to examine the structure of the intestinal. Samples of intestinal tissue were fixed, dehydrated, cleared, embedded in paraffin, sectioned, and strained. Picture were taken at a 100× zoom level with an Olympus microscope (Olympus, Richmond Hill, ON, Canada). Five fields were randomly selected for analysis of villus height and crypt depth in each cross-section, and the average of these measurements was determined for every sample.

### Determination of immune organs indices

To determine the organ index, the weight of immune organs such as spleen, thymus, and bursa of fabricius were measured and calculated using the formula: organ index = (organ weight/live weight of bird)×100%.

### Serum analysis

Diamine oxidase (DAO) activity and the concentrations of *D*-lactic acid (*D*-LA), endotoxin (ET), tumor necrosis factor-α (TNF-α), interleukin-1β (IL-1β), IL-6, immunoglobulin A (IgA), IgG and IgM in serum were measured with specific chicken ELISA kits, following the guidelines provided by the manufacturer (Nanjing Jiancheng Bioengineering Institute, Jiangsu, China).

### Measurement of tight junction proteins and cytokines in intestinal tissue

Total RNA from the ileal mucosa was extracted using RNA extraction kit (Invitrogen, Carlsbad, CA, USA). RNA quality and quantity were assessed using the RNA 6000 Nano Lab Chip kit in a bioanalyzer (Agilent Technologies, Shanghai, China). Total RNA (2 ng) was reverse transcribed into cDNA using reverse transcription (Bioteke Corporation, Beijing, China). cDNA was amplified by real-time polymerase chain reaction (PCR) using SYBR qPCR SuperMix (TransGen Biotech, Beijing, China). Supplement 1 contains the primers for *TNF-α, IL-1β, IL-6, Claudin-1, Occludin*, and zonula occludens-1 (*ZO-1*). The glyceraldehyde-3-phosphate dehydrogenase (*GAPDH*) gene was used as internal control.

### Detection of gut microbiota

We conducted microbiome analysis on intestinal content samples using the method previously outlined by Zhou et al [16] through 16S rRNA gene sequencing. Following the extraction of DNA from the entire bacterial community and assessment of its quality and quantity, the V3–V4 region of 16S rRNA was amplified with 515F GTGCCAGCMGCCGCGG TAA and 806R GGACTACHVGGGTWTCTAAT primers. The PCR products were analyzed using gel electrophoresis, purified with a Qiagen Gel Extraction Kit (Qiagen, Hilden, Germany) to eliminate unwanted DNA fragments, and evaluated with a Qubit@2.0 Fluorometer (Thermo Scientific, Waltham, MA, USA) and Agilent Bioanalyzer 2100 system. Finally, the products were combined evenly and then processed on an Illumina NovaSeq platform to produce 250 bp paired-end reads.

After merging and filtering the raw reads, they were ultimately grouped into operational taxonomic units (OTUs). The MUSCLE software (Version 3.8.31, https://drive5.com/muscle) was utilized for conduction multiple sequence alignment. QIIME (version 1.9.1) was used to calculate all the indices of alpha diversity such as Chao 1, Shannon, Simpson, Observed-species, and Good-coverage, as well as beta diversity analysis. The results were then visualized using R software (version 2.15.3, http://www.r-project.org).

### Assessment of short-chain fatty acids

Short-chain fatty acids (SCFA) in intestinal content samples were determined by gas chromatography-mass spectrometry (GC-MS) analysis. Initially, 50 mg of the intestinal content was vortexed in 1mL of water, then homogenized in a ball mill for 4 min at 40 Hz, treated with ultrasound (three times for 5 min each, placed in ice water), and finally centrifuged (for 20 min at 13,800×g, at 4°C). After transferring 0.8 mL of supernatants to a new 2 mL Eppendorf tube, 0.1 mL of 50% sulfuric acid and 0.8 mL of extraction solution (1 mL methyl tert-butyl ether with 25 μg 2-methylvaleric acid as internal standard) were added. The mixture was the vortexed for 10 s, oscillated for 10 min, treated with ultrasound for 10 min (incubated in ice water), centrifuged for 15 min at 13,800×g and 4°C, and finally stored for 30 min at −20°C. The supernatants were moved to a new 2 mL glass container for GC-MS vials.

Serial dilution of a standard stock solution was used to establish the linear range of the analytical test. The linearity range was from 0.02 to 1 mg/L for all SCFAs except acetic acid (0.2 to 10 mg/L).

A Shimadzu GC2030-QP2020 NX gas chromatograph–mass spectrometer (Shimadzu, Kyoto, Japan) coupled to an HP-FFAP capillary column (Agilent, Folsom, CA, USA) was used for GC-MS analysis. The analyte was injected in 1 μL aliquot under 5:1 split mode. Helium served as the carrier gas, with a front inlet purge flow of 3 mL/min and a column gas flow rate of 1 mL/min. The starting temperature was maintained at 80°C for 1 min, then elevated to 200°C with an increment of 10°C/min for 5 min, and finally escalated to 240°C with an increase of 40°C/min for 1 min. The temperatures of the injection, transfer line, quad, and ion source were set 240°C, 240°C, 150°C, and 200°C, respectively. In electron impact mode, the energy was recorded as −70 eV. Mass spectrometry data were obtained using scan/selective ion monitoring mode with an m/z range of 33 to 150 following a 3.5 min solvent delay.

### Neurotransmitter and hormone analysis

Intestinal content, serum and tissue samples from the cerebellum, hypothalamus and kidney were lysed by adding 80% methanol aqueous solution. After being homogenized, the samples were spun at 10,000×g for 10 min at 4°C, and the supernatant was gathered. Then, transfer 200 μL of the supernatant to a new tube and mix with 800 μL of cold acetonitrile (which includes the internal standards). Following vortexing, the samples underwent centrifugation at 10,000×g for 10 min at 4°C, after which the resulting supernatant was used for instrumental analysis. In addition, adrenocorticotropin (ACTH) was detected qualitatively using the method of external standards and semi-quantitatively by calculating the peak area. Absolute quantification of all other compounds was performed according to the internal standard method. Internal standards and their concentrations included acetylcholine-d9 (50 ng/mL), serotonin-d4 (1,000 ng/mL), epinephrine (EN)-d6 (1,000 ng/mL), norepinephrine (NE)-d6 (1,000 ng/mL), and cortisol-d4 (1,000 ng/mL).

The examination was conducted using the UPLC system (ExionLC; Sciex, Framingham, MA, USA) connected to a triple quadrupole mass spectrometer (Triple Quad 6500; Sciex). Detection was performed in MRM positive ion mode. The samples were divided by utilizing an Atlantis HILIC column (4.6 mm ×100 mm, i.d.: 3 μm; Waters Corporation, Milford, MA, USA). The aqueous mobile phase A consisted of water with 0.1% formic acid and 5 mM ammonium formate, while the organic mobile phase B was composed of acetonitrile with 0.1% formic acid. The flow rate was set at 0.4 mL/min and the injection volume was 5 μL. The ion source settings included an ion spray voltage of 5,500 V, a source temperature of 550°C, a curtain gas pressure of 35 psi, and ion source gas 1 (nebulizer) and 2 (turbo ion spray) pressures of 55 psi.

### Statistical analysis

The experimental units consisted of five replicates. SPSS 23.0 (IBM-SPSS Inc., Chicago, IL, USA) was used for statistical analysis through one-way analysis of variance (ANOVA). The results in the tables were presented as means and pooled standard error of the mean, and standard deviation in figures. Tukey’s multiple comparison test was used to assess notable discrepancies among treatments at a significance level of p<0.05.

## RESULTS

### Effects of pellitorine, vitexin 2-O-rhamnoside, and vitexin 2-O-rhamnoside+pellitorine on growth performance of chickens

Table 2 displays the growth performance statistics for the chickens. Chickens that received the VR+PT supplemented diet showed the highest final weight and ADG (p<0.05) when compared to chickens in the CON and PSE groups. And the F/G in the VR+PT group was the lowest (p<0.05).

### Effects of pellitorine, vitexin 2-O-rhamnoside, and vitexin 2-O-rhamnoside+pellitorine on the intestinal mucosal barrier of chickens

**Figure 1:**
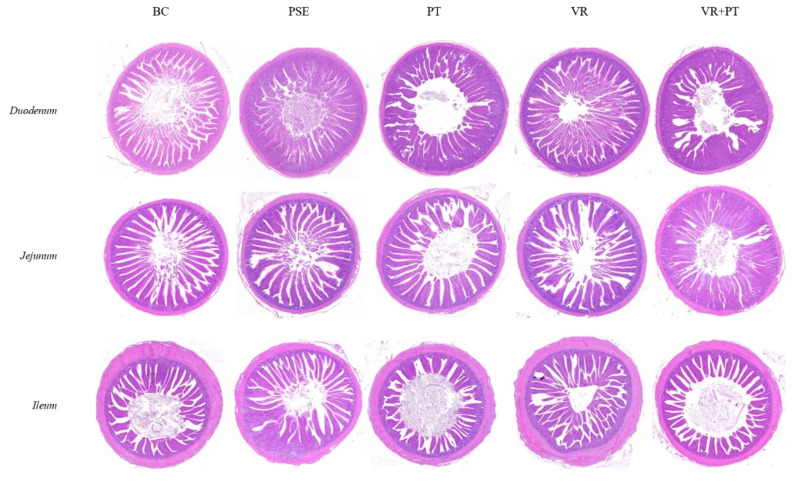
HE staining map of the duodenum, jejunum, and ileum (×100). CON group, basal diet; PSE group, basal diet+200 mg/kg PSE; PT group, basal diet+0.563 mg/kg PT; VR group, basal diet+1.321 mg/kg VR; VR+PT group, basal diet+1.321 mg/kg VR+0.563 mg/kg PT. PSE, *Piper sarmentosum* extract; PT, pellitorine; VR, vitexin-2-O-rhamnoside; SD, standard deviation; ANOVA, analysis of variance.

Table 3 and Figure 1 demonstrate that PSE, PT, and VR+PT reduced the depth of the duodenum crypts and increased height of the villi and the V/C ratio of the duodenum in comparison to the CON group (p<0.05). Additionally, PSE and PT raised the height of the villus and the V/C ratio of the jejunum compared to the CON group (p<0.05). PSE and VR decreased the depth of the crypt in the ileum and inproved the V/C ratio in the ileum (p<0.05). After 35 days of treatment with PT, VR, or VR+PT in this experiment, the serum *D*-LA levels were lower compared to the CON and PSE group (p<0.05) (Table 4). Compared to the CON group, VR and PT individually increased the mRNA levels of Occludin and Claudin-1 (p<0.05), as shown in Table 5. Meanwhile, VR+PT raised the levels of ZO-1 and Claudin-1 expression in comparison to the CON group (p<0.05) (Table 5), showing no notable distinction when compared to the PSE group (p>0.05). The findings suggest that the joint use of VR and PT has a similar impact as PSE in enahncing the intestinal barrier function of chickens.

### Effects of pellitorine, vitexin 2-O-rhamnoside, and vitexin 2-O-rhamnoside+pellitorine on immune function of chickens

The immune organ indices are shown in Table 6. Among all groups, chickens in the VR+PT group had the highest immune organ indices (liver, thymus, bursa of fabricius, and spleen) (p<0.05).

Tables 4, 5 display the impact of various treatments on the concentrations of immune-related substances in the serum and ileal mucosa of chickens. ELISA tests revealed that the serum levels of the proinflammatory factors IL-1β and IL-6 were lower in the PSE and VR+PT groups compared to the CON group (p<0.05) (Table 4). Consistently, PCR analyses revealed similar results (Table 5). Additionally, the PT and VR+PT groups exhibited elevated serum IgM levels (p<0.05) compared to the CON group as shown in Table 4.

### Effects of pellitorine, vitexin 2-O-rhamnoside, and vitexin 2-O-rhamnoside+pellitorine on gut microbial communities

**Figure 2:**
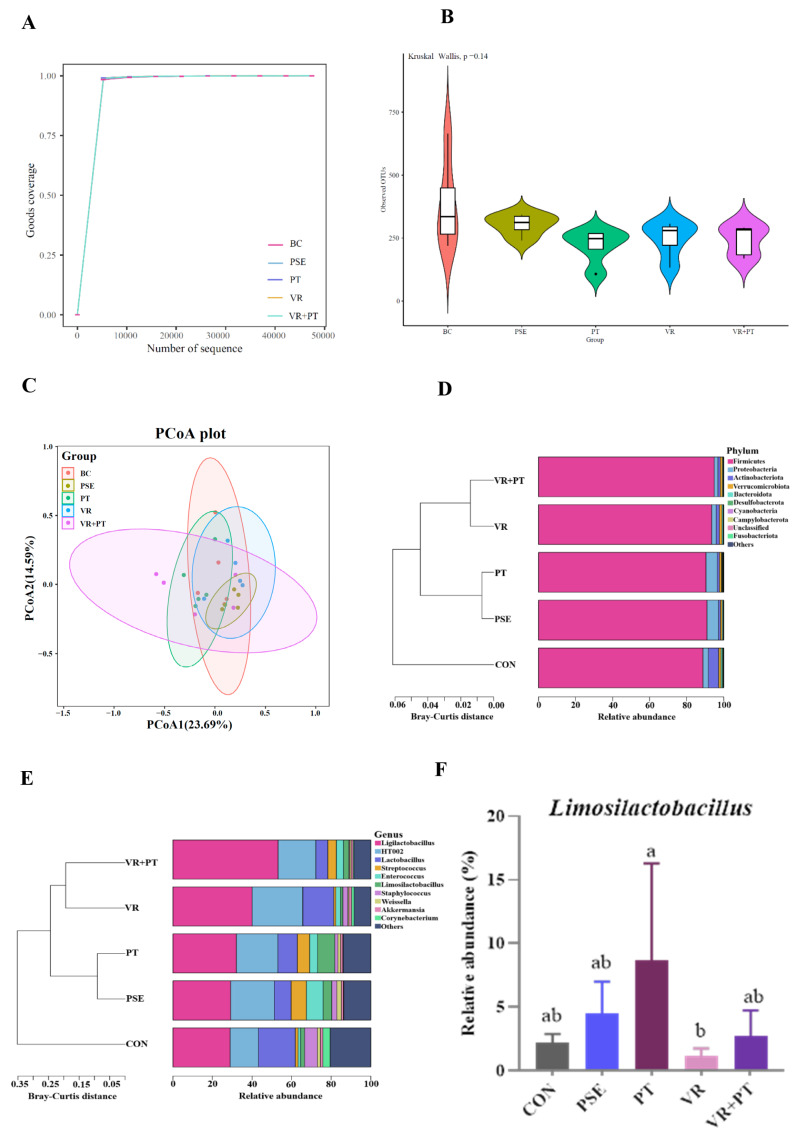
Intestinal microbiota diversity and composition. (A,B) Index of the goods coverage and observed OUTs for alpha diversity of each group, respectively. (C) The PCoA plot of bacterial beta-diversity based on the Bray-Curtis dissimilarity index from all groups. (D,E) Comparison of relative percentage abundance of taxa (within the top 10 and others) among all groups at the phylum and genus levels, respectively. The hierarchical cluster tree based on Bray-Curtis distance is at the left of each plot, and the stacked bar chart of mean relative taxa abundance is at the right of each plot. (F) Relative abundances of genus *Limosilactobacillus* among all groups. CON group, basal diet; PSE group, basal diet +200 mg/kg PSE; PT group, basal diet+0.563 mg/kg PT; VR group, basal diet+1.321 mg/kg VR; and VR+PT group, basal diet+1.321 mg/kg VR+0.563 mg/kg PT. Data are presented as mean ± SD. Different letters within the figure indicate significant differences (ANOVA; p<0.05; n = 5). PSE, Piper sarmentosum extract; PT, pellitorine; VR, vitexin-2-O-rhamnoside; PCoA, principal coordinate analysis; OTUs, operational taxonomic units; SD, standard deviation; ANOVA, analysis of variance.

We evaluated the alpha-(within-sample) and beta-(between samples) diversity indices at two different levels to analyze the species richness of gut microbial using 16S rRNA gene amplicon sequencing. For alpha-diversity metrics, Good’s coverage and OTUs of microbial communities were determined. The rarefaction curves were generated to demonstrate that each sample was sequenced deeply enough to reach its maximum diversity level, as indicated by a Good’s coverage index exceeding 0.99 (see Figure 2A). No notable variances were observed in the quantity of OTUs among the groups (p>0.05) (Figure 2B). Furthermore, beta-diversity was represented through principal coordinate analysis (PCoA) with Bray-Curtis dissimilarity distances, where PCo1 and PCo2 explained 23.69% and 14.59% of the total variance, correspondingly (Figure 2C).

The microbial composition of each group showed changes at various taxonomic levels (phylum, class, order, and family) as shown in Supplement 2, but these differences were not statistically significant. From the hierarchical cluster tree based on Bray-Curtis distance, similar bacterial structures were found between the VR and VR+PT groups, and between the PSE and PT groups at the phylum and genus levels, respectively (Figures 2D, 2E). At the phylum level, the CON group exhibited decreased levels of *Firmicutes* and increased levels of *Actinobacteriota* compared to other groups, however, these variances did not reach statistical significance (p>0.05) (Figure 2D). Additionally, in terms of genus, *Ligilactobacillus* and *HT002* were relatively less abundant while *Lactobacillus* was relatively more abundant in the CON group compared to the other four groups (p>0.05) (Figure 2E). The PT group had a greater proportion of *Limosilactobacillus* at the genus level compared to the VR group (p<0.05), however, there were no significant differences in relative abundance between the VR+PT and PT groups (p>0.05) (Figure 2F).

### Effects of pellitorine, vitexin 2-O-rhamnoside, and vitexin 2-O-rhamnoside+pellitorine on short-chain fatty acid levels in intestinal contents

SCFAs, with fewer than six carbon atoms, are the primary secondary metabolites produced by the fermentation of undigestible foods by gut bacteria, impacting the body’s function. Table 7 displays that there were no notable variations in the concentrations of acetic acid, propionic acid, isobutyric acid, butyric acid, isovaleric acid, valeric acid, and total SCFAs across the groups (p>0.05).

### Effects of pellitorine, vitexin 2-O-rhamnoside, and vitexin 2-O-rhamnoside+pellitorine on neurotransmitter and hormone levels of chickens

We investigated the ACTH, acetylcholine, serotonin, EN, NE, and cortisol levels in the hypothalamus, cerebellum, intestinal contents, serum, and kidney (Supplements 3, 4).

**Figure 3:**
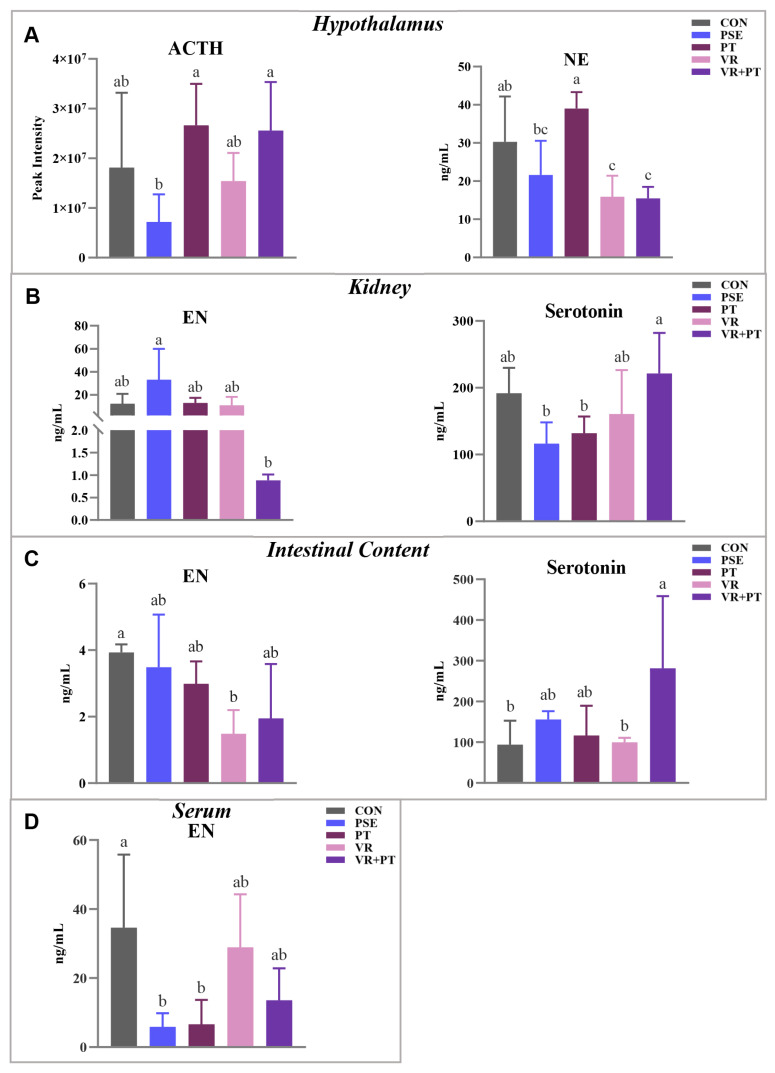
Effects of different treatments on hormone levels detected in different tissues of chickens. (A) ACTH and NE levels in the hypothalamus; (B) and (C) the levels of EN and serotonin in the kidney and intestinal content, respectively; (D) EN level in serum. Data are presented as mean±SD. Different letters within the figure indicate significant differences (ANOVA; p<0.05; n = 5). ACTH, adrenocorticotropic hormone; NE, norepinephrine; PSE, *Piper sarmentosum* extract; PT, pellitorine; VR, vitexin-2-O-rhamnoside; EN, epinephrine; SD, standard deviation; ANOVA, analysis of variance.

Figure 3A illustrates that the ACTH concentration in the hypothalamus was elevated in the PT and VR+PT groups comparted to the CON grous (p<0.05), while the NE level in the VR and VR+PT groups was reduced (p<0.05). Furthermore, the VR+PT group had the lowest EN level in the kidney (p<0.05) as shown in Figure 3B, while the serotonin level in the kidney was the highest in the same group (p<0.05) according to Figure 3B. Moreover, the concentration of serotonin in the intestinal contents of the VR+PT group exceeded that of the CON group (Figure 3C). The serum EN level was lower in the other groups comparted to the CON group (p<0.05) (Figure 3D). In particular, the VR, PT, and VR+PT groups tended to show lower EN levels in both intestinal contents and serum compared to the CON group (Figures 3C, 3D).

## DISCUSSION

Growth performance is a key indicator that directly reflects the health status of poultry [17]. The findings indicated that the VR group had notably greater final weight and ADG compared to the PSE group, suggesting that VR is likely a key active ingredient in PSE. The positive impact on growth performance may be due to its strong ability to scavenge free radicals and act as an antioxidant [18,19]. The study also found that neither PT nor VR alone had a significant impact on the growth of chickens compared to the CON group. However, the final weight and ADG of chickens were notably higher in the VR+PT group, suggesting that the combination of PT and VR is more effective in enhancing growth performance than using PT or VR separately. PT contains a single hydrocarbon chain with a single polar amide group, possessing amphiphilic properties. Therefore, it was speculated that PT may act as a surfactant to improve the intestinal absorption and bioavailability of VR, and this process may enhance its role in improving growth performance of chickens. Moreover, it has been reported that both VR and PT have anti-inflammatory activity [18,20], and presumably, combined administration of both has the potential to exert synergistic anti-inflammatory effects, increasing the synthesis of proteins in chickens by synergistically modulating the target of rapamycin pathway [21].

The intestinal mucosal barrier is a selective semipermeable membrane that is not only responsible for nutrient uptake and bidirectional gut-brain interaction but also serves as a crucial barrier against many pathogens [22]. Typically, the morphological structure of the intestines can provide insight into the body’s ability to digest and absorb nutrients. This is commonly assessed by examining the height of the villi, the depth of the crypts, and the ratio of villus height to crypt depth [23]. Additionally, claudin-1, occludin, and ZO-1 are viewed as crucial elements of the paracellular permeability barrier in the intestinal epithelium [24]. The serum levels of DAO, *D*-LA, and ET are frequently utilized as indirect indicators for assessing intestinal permeability and barrier function [25]. The study found that adding VR, PT, or both, to the diet enhanced the intestinal structure and regulated tight junction proteins in the ileum to different degrees. It is speculated that these variations may be due to the distinct chemical characteristics of VR and PT, or their combined effects. Nevertheless, some interesting commonalities were also observed. Morphometrically, the VR, PT, and VR+PT groups showed better intestinal morphology and a higher intestinal absorption capacity than the CON group. At the same time, there was an increase in the expression of *ZO-1, Occludin*, and *Claudin-1* in the ileum, along with a reduction in the serum levels of *D*-LA in the VR, PT, and VR+PT groups. Past studies have indicated that polyphenols and amides can potentially lessen oxidative stress and inhibit inflammatory responses, leading to improved gut health in animals [26,27]. As expected, VR, PT, and VR+PT can enhance digestion and nutrient absorption and strengthen the intestinal mucosa barrier, thus promoting gut health of chickens.

Changes in immune organ indices can be used to evaluate the degree of immune organ development, which is positively correlated with immunity in poultry [28]. Our study revealed that the organ indices of the liver, thymus, and spleen in chickens were higher in the VR+PT group, but not in the VR and PT groups, than in the CON group, suggesting that VR and PT play a synergistic role in the improvement of the immune function of developing immune organs. Furthermore, inflammatory cytokines like TNF-α, IL-1β, and IL-6 play a crucial role in cellular immune reactions and are typically released in the initial stages of both acute and chronic inflammatory conditions [29]. It is worth noting that the concentrations of IL-1β and IL-6 in the blood were notably reduced in the PSE and VR+PT groups compared to the CON group. PCR analysis yielded similar results, showing a significant decrease in *IL-1β* and *IL-6* mRNA levels in the ileal mucosa of the PSE and VR+PT groups compared to the CON group. Indeed, such results further implied that VR and PT may be two of the main pharmacodynamic component of PSE exerting anti-inflammatory activity. Studies has demonstrated that VR and PT can effectively reduce inflammation by impacting the PI3K/AKT and NF-κB signaling pathways [18,20]. These observations led to the speculation that the interaction between the PI3K/AKT and NF-κB pathways could serve as a crucial communication network for the anti-inflammatory benefits of the combining VR and PT. However, this hypothesis requires experimental validation.

Surprisingly, we found that the combined supplementation of VR and PT can positively abrogate some negative consequences of PT or VR alone on organismal fitness. In particular, our findings showed a notable distinction between the VR and PT groups, with the PT group having a greater relative abundance of *Limosilactobacillus* compared to the VR group. Likewise, the study indicated a notable increase in hypothalamic NE levels in the PT group compared to the VR group. However, notably, the relative abundance of *Limosilactobacillus* in the VR+PT group was intermediate between that of the VR and PT groups but not significantly different from either. Simultaneously, the levels of NE in the hypothalamus were similar in both the VR and VR+PT groups. According to the reclassification of genus *Lactobacillus* in 2020 [30], *Limosilactobacillus*, a novel lactobacillus genus, has been shown to possess probiotic properties, reducing inflammation and inhibiting bacterial translocation [31,32]. According to reports, NE, a neurotransmitter in the stress-related catecholamines category, is released by the sympathetic nervous system that can trigger the release of inflammatory cytokines by activating the NF-κB signaling pathway [33,34]. These observations support the synergistic beneficial effects of VR and PT on the bidirectional communication that occurs between microbiota and the brain.

Serotonin, also known as 5-hydroxytryptamine, is a neurotransmitter produced mainly in the enterochromaffin cells of the small intestine from tryptophan, as widely recognizes [35]. Serotonin, a crucial signaling molecule, facilitates two-way communication between the gut and the brain and is essential for regulating various bodily functions such as gut movement, enzymes release, nutrient uptake, and immune response [36,37]. The findings suggest that that the VR+PT group had a notably elevated level of serotonin in the intestines compared to the CON group, while the PSE, VR, and PT groups did not show any significant differences in intestinal serotonin level when compared to the CON group. This confirms that the gut-brain axis could be an important target for VR and PT, which exert synergistic beneficial effects.

## CONCLUSION

Overall, this study demonstrated that VR (1.321 mg/kg in diets) and PT (0.563 mg/kg in diets) could synergistically promote gut health of chickens via improving intestinal mucosal morphology, strengthening the intestinal barrier function, and inhibiting the inflammatory response; these effects are comparable to those of PSE (200 mg/kg in diets) supplementation alone. Preliminary confirmation shows that VR and PT are two of the main active ingredients in PSE that synergistically regulate intestinal health.

## Figures and Tables

**Table 1:** Ingredient and nutrient levels of basal diet (%, air-dry basis)

Items	Contents
Ingredients	100.00
Corn	60.00
Soybean meal	35.50
Limestone	1.70
Calcium hydrogen phosphate	1.30
Premix^1)^	1.50
Nutrient levels^2)^	
Metabolic energy (MJ/kg)	12.01
Crude protein	21.00
Lysine	1.11
Methionine	0.53
Calcium	1.05
Total phosphorus	0.58

1) Premix provide the following per kilogram of diet: VA 11,000 IU, VE 20 mg, VK 3 mg, VD 3,000 IU, VB1 2 mg, VB2 8 mg, VB12 0.04 mg, Fe 65 mg, Cu 10 mg, Mn 77 mg, Zn 70 mg, pantothenic acid 19 mg, folic acid 1.1 mg.

2) The nutrient levels are calculated values.

**Table 2:** Effect of different treatment on the growth performance of chickens

Item	Groups^1)^					SEM	p-value

	CON	PSE	PT	VR	VR+PT		
Initial weight (g)	26.07	26.43	25.83	25.63	26.35	0.30	0.305
Final weight (g)	229.83^bc^	223.30^c^	239.54^abc^	257.93^ab^	261.53^a^	7.07	0.005
ADG (g)	5.81^bc^	5.63^c^	6.10^abc^	6.63^ab^	6.73^a^	0.20	0.004
ADFI (g)	14.75	15.11	16.92	15.99	14.93	0.52	0.058
F/G	2.54^ab^	2.70^ab^	2.78^a^	2.42^ab^	2.22^b^	0.11	0.023

1) n = 5.

a–c In the same row, values with no letter or the same letter superscripts mean no significant difference (p>0.05), while with different letter superscripts mean significant difference (p<0.05).

CON, chickens were given a basal diet; PSE, *Piper sarmentosum* extract; PT, pellitorine; VR, vitexin-2-O-rhamnoside; SEM, standard error of the mean; ADG, average daily gain; ADFI, average daily feed intake; F/G, the ratio of average daily feed intake to average daily gain.

**Table 3:** Effect of different treatment on intestinal structure of chickens

Item	Groups^1)^					SEM	p-value

	CON	PSE	PT	VR	VR+PT		
Duodenum							
Villus height (μm)	918.01^c^	1,084.55^b^	1,199.72^a^	952.24^c^	1,079.74^b^	2.41	<0.001
Crypt depth (μm)	204.92^a^	127.01^c^	171.73^b^	165.61^b^	149.23^c^	2.99	<0.001
V/C	4.96^c^	8.67^a^	7.06^b^	5.93^bc^	7.20^ab^	0.15	<0.001
Jejunum							
Villus height (μm)	715.24^b^	858.79^a^	847.16^a^	729.10^b^	748.83^ab^	12.07	0.002
Crypt depth (μm)	152.34	126.54	133.51	143.01	144.03	2.11	0.081
V/C	4.71^c^	6.76^a^	6.36^ab^	5.50^bc^	5.33^bc^	0.14	<0.001
Ileum							
Villus height (μm)	418.29	458.22	567.28	501.77	469.90	15.84	0.084
Crypt depth (μm)	137.74^a^	95.14^bc^	127.98^ab^	85.16^c^	115.75^abc^	5.09	0.001
V/C	3.19^c^	4.70^b^	3.87^c^	5.91^a^	3.75^c^	0.05	<0.001

1) n = 5.

a–c In the same row, values with no letter or the same letter superscripts mean no significant difference (p>0.05), while with different letter superscripts mean significant difference (p<0.05).

CON, chickens were given a basal diet; PSE, *Piper sarmentosum* extract; PT, pellitorine; VR, vitexin-2-O-rhamnoside; SEM, standard error of the mean; V/C, the ratio of villus height to crypt depth.

**Table 4:** The levels of DAO, *D*-LA, ET, cytokines and immunoglobulins in the serum of chickens among the different groups

Item	Groups^1)^					SEM	p-value

	CON	PSE	PT	VR	VR+PT		
DAO (pg/mL)	14.55	13.98	14.43	13.98	14.49	0.31	0.534
*D*-LA (nmol/mL)	211.33^a^	193.86^a^	134.81^b^	124.67^b^	118.17^b^	10.26	<0.001
ET (nmol/mL)	62.35	59.77	60.40	61.85	63.10	1.09	0.203
TNF-α (pg/mL)	60.61^a^	56.65^ab^	56.55^ab^	53.18^b^	56.79^ab^	1.41	0.027
IL-1β (pg/mL)	526.81^a^	468.96^b^	504.87^ab^	481.92^b^	479.93^b^	9.03	0.001
IL-6 (pg/mL)	23.51^a^	19.27^b^	20.59^b^	21.30^ab^	20.80^b^	0.57	0.001
IgA (μg/mL)	223.39	231.04	223.39	235.72	240.39	6.14	0.241
IgG (μg/mL)	1,847.17	1,760.91	1,919.06	1,890.30	1,851.28	51.66	0.291
IgM (μg/mL)	568.05^ab^	537.70^b^	640.90^a^	610.54^ab^	619.04^a^	24.42	0.048

1) n = 5.

a,b In the same row, values with no letter or the same letter superscripts mean no significant difference (p>0.05), while with different letter superscripts mean significant difference (p<0.05).

DAO, diamine oxidase; D-LA, D-lactic acid; ET, endotoxin; CON, chickens were given a basal diet; PSE, *Piper sarmentosum* extract; PT, pellitorine; VR, vitexin-2-O-rhamnoside; SEM, standard error of the mean; TNF-α, tumor necrosis factor-α; IL-1β, interleukin-1β; IL-6, interleukin-6; IgA, immunoglobulin A; IgG, immunoglobulin G; IgM, immunoglobulin M.

**Table 5:** Relative mRNA expression of cytokines, and tight junction proteins in the ileal mucosa of chickens among the different groups

Item	Groups^1)^					SEM	p-value

	CON	PSE	PT	VR	VR+PT		
TNF-α	1.00	0.84	0.88	0.67	0.81	0.09	0.188
IL-1β	1.00^a^	0.68^b^	0.88^ab^	0.74^ab^	0.72^b^	0.06	0.010
IL-6	1.00^a^	0.58^b^	0.61^b^	0.64^b^	0.60^b^	0.02	<0.001
ZO-1	1.00^b^	1.40^ab^	1.14^b^	1.38^ab^	1.68^a^	0.11	0.003
Occludin	1.00^b^	1.16^ab^	1.09^ab^	1.41^a^	1.30^ab^	0.09	0.039
Claudin-1	1.00^c^	1.44^ab^	1.57^a^	1.21^bc^	1.71^a^	0.07	<0.001

1) n = 5.

a,b In the same row, values with no letter or the same letter superscripts mean no significant difference (p>0.05), while with different letter superscripts mean significant difference (p<0.05).

CON, chickens were given a basal diet; PSE, *Piper sarmentosum* extract; PT, pellitorine; VR, vitexin-2-O-rhamnoside; SEM, standard error of the mean; TNF-α, tumor necrosis factor-α; IL-1β, interleukin-1β; IL-6, interleukin-6; ZO-1, zonula occludens-1.

**Table 6:** The immune organ index of chickens among different groups

Item (%)	Groups^1)^					SEM	p-value

	CON	PSE	PT	VR	VR+PT		
Liver index	2.30^b^	2.48^ab^	2.19^b^	2.17^b^	2.99^a^	0.13	0.001
Thymus index	0.48^b^	0.73^ab^	0.59^ab^	0.59^ab^	0.83^a^	0.06	0.004
Bursa of fabricius index	0.31^ab^	0.30^ab^	0.33^ab^	0.25^b^	0.58^a^	0.07	0.049
Spleen index	0.22^b^	0.36^ab^	0.30^ab^	0.22^b^	0.58^a^	0.08	0.020

1) n = 5.

a,b In the same row, values with no letter or the same letter superscripts mean no significant difference (p>0.05), while with different letter superscripts mean significant difference (p<0.05).

CON, chickens were given a basal diet; PSE, *Piper sarmentosum* extract; PT, pellitorine; VR, vitexin-2-O-rhamnoside; SEM, standard error of the mean.

**Table 7:** Total SCFAs concentration and SCFAs profile in intestinal contents of chickens among the different groups

Item	Groups (μg/g)^1)^					SEM	p-value

	CON	PSE	PT	VR	VR+PT		
Acetic acid	23.54	28.66	31.62	24.14	23.35	5.35	0.751
Propionic acid	0.63	1.68	0.74	0.68	0.49	0.41	0.295
Isobutyric acid	0.92	0.91	0.93	0.85	0.89	0.04	0.648
Butyric acid	0.20	0.22	0.25	0.18	0.39	0.07	0.324
Isovaleric acid	0.06	0.07	0.20	0.04	0.09	0.05	0.163
Valeric acid	0.09	0.08	0.12	0.11	0.16	0.02	0.078
SCFAs	25.43	31.61	33.87	26.00	25.36	5.55	0.722

1) n = 5.

In the same row, values with no letter or the same letter superscripts mean no significant difference (p>0.05), while with different letter superscripts mean significant difference (p<0.05).

CON, chickens were given a basal diet; PSE, *Piper sarmentosum* extract; PT, pellitorine; VR, vitexin-2-O-rhamnoside; SEM, standard error of the mean; SCFAs, short-chain fatty acids.

## SUPPLEMENTARY MATERIAL

**Supplement 1**. Primer sequences used in the current study

**Supplement 2**. Relative abundance of the top 10 gut microbiota from the five groups at different levels

**Supplement 3**. Neurotransmitter and hormone levels in different tissues in the different groups (ng/mL)

**Supplement 4**. Relative ACTH content in serum, intestinal contents, and different tissues (hypothalamus, cerebellum, and kidney) of each experimental group, as quantified by UPLC-QQQ-MS.
