# Supplementary material for: The impact of active components from Piper sarmentosum on the growth, intestinal barrier function, and immunity of broiler chickens
Source: Anim Biosci. 2025 Feb 27;38(7):1522–34. doi: 10.5713/ab.24.0736 (PMC12229918; doi:10.5713/ab.24.0736)
Supplement: Supplementary file 1 [file ab-24-0736-Supplementary-1.pdf]

Supplement 1. Primer sequences used in the current study

Gene	Accession No.	Primer sequences (5'→3')	Product size (bp)
GAPDH	NM_204305.1	F: ACTGTCAAGGCTGAGAACGG<br>R: ACCTGCATCTGCCCATTTGA	151
IL-1β	XM_015297469.1	F: CTTCCTTCCAGCGCTCCTT<br>R: CCGTAGAAGGTCTCTTCGCT	189
IL-6	NM_204628.2	F: CTCGTCCGGAACAACCTCAA<br>R: GGAGAGCTTCGTCAGGCATT	96
TNF-α	NM_204267	F: TTCCTGCTGGGGTGCATAG<br>R: AAGAACCAACGTGGGCATTG	106
ZO-1	NM_015278981.2	F: TCTGAACCCGTTAGGGAGGAT<br>R: CTGTATACCGGCTGAGAAGCA	143
Occludin	NM_205128.1	F: AGACGCGCAGTAAGATCTGG<br>R: CACGTTCTTCACCCACTCCT	104
Claudin-1	NM_001013611.2	F: ACCCGTTAACACCAGATTTGAG<br>R: TGGGTAGGATGTTTCACTCCG	124

GAPDH, glyceraldehyde-3-phosphate dehydrogenase; *IL-1β*, Interleukin 1β; *IL-6*, Interleukin 6; *TNF-α*, Tumor necrosis factor α; *ZO-1*, zonula occludens-1.

Supplement 2. Relative abundance of the top 10 gut microbiota from the five groups at different levels

Name	Groups					SEM	*P*
	CON	PSE	PT	VR	VR+PT		
At the phylum level							
Firmicutes	88.77	90.94	90.50	93.53	94.88	3.51	0.745
Proteobacteria	3.01	6.25	5.98	2.42	2.03	2.75	0.708
Actinobacteriota	5.51	1.10	1.22	1.73	1.27	1.80	0.387
Verrucomicrobiota	1.29	0.93	1.17	1.42	1.06	0.22	0.551
Bacteroidota	0.75	0.42	0.46	0.53	0.39	0.15	0.488
Desulfobacterota	0.32	0.21	0.21	0.25	0.20	0.05	0.443
Cyanobacteria	0.21	0.04	0.09	0.02	0.03	0.09	0.570
Campylobacterota	0.05	0.07	0.10	0.04	0.07	0.03	0.686
unclassified	0.00	0.00	0.22	0.01	0.01	0.07	0.120
Fusobacteriota	0.02	0.01	0.03	0.03	0.00	0.02	0.817
Others	0.06	0.04	0.03	0.03	0.04	0.01	0.628

At the class level
