# Supplementary material for: The impact of active components from Piper sarmentosum on the growth, intestinal barrier function, and immunity of broiler chickens
Source: Anim Biosci. 2025 Feb 27;38(7):1522–34. doi: 10.5713/ab.24.0736 (PMC12229918; doi:10.5713/ab.24.0736)
Supplement: Supplementary file 2 [file ab-24-0736-Supplementary-2.pdf]

Supplement 1. Primer sequences used in the current study

Gene	Accession No.	Primer sequences (5'→3')	Product size (bp)
GAPDH	NM_204305.1	F: ACTGTCAAGGCTGAGAACGG<br>R: ACCTGCATCTGCCCATTTGA	151
IL-1β	XM_015297469.1	F: CTTCCTTCCAGCGCTCCTT<br>R: CCGTAGAAGGTCTCTTCGCT	189
IL-6	NM_204628.2	F: CTCGTCCGGAACAACCTCAA<br>R: GGAGAGCTTCGTCAGGCATT	96
TNF-α	NM_204267	F: TTCCTGCTGGGGTGCATAG<br>R: AAGAACCAACGTGGGCATTG	106
ZO-1	NM_015278981.2	F: TCTGAACCCGTTAGGGAGGAT<br>R: CTGTATACCGGCTGAGAAGCA	143
Occludin	NM_205128.1	F: AGACGCGCAGTAAGATCTGG<br>R: CACGTTCTTCACCCACTCCT	104
Claudin-1	NM_001013611.2	F: ACCCGTTAACACCAGATTTGAG<br>R: TGGGTAGGATGTTTCACTCCG	124

GAPDH, glyceraldehyde-3-phosphate dehydrogenase; *IL-1β*, Interleukin 1β; *IL-6*, Interleukin 6; *TNF-α*, Tumor necrosis factor α; *ZO-1*, zonula occludens-1.

Supplement 2. Relative abundance of the top 10 gut microbiota from the five groups at different levels

Name	Groups					SEM	*P*
	CON	PSE	PT	VR	VR+PT		
At the phylum level							
Firmicutes	88.77	90.94	90.50	93.53	94.88	3.51	0.745
Proteobacteria	3.01	6.25	5.98	2.42	2.03	2.75	0.708
Actinobacteriota	5.51	1.10	1.22	1.73	1.27	1.80	0.387
Verrucomicrobiota	1.29	0.93	1.17	1.42	1.06	0.22	0.551
Bacteroidota	0.75	0.42	0.46	0.53	0.39	0.15	0.488
Desulfobacterota	0.32	0.21	0.21	0.25	0.20	0.05	0.443
Cyanobacteria	0.21	0.04	0.09	0.02	0.03	0.09	0.570
Campylobacterota	0.05	0.07	0.10	0.04	0.07	0.03	0.686
unclassified	0.00	0.00	0.22	0.01	0.01	0.07	0.120
Fusobacteriota	0.02	0.01	0.03	0.03	0.00	0.02	0.817
Others	0.06	0.04	0.03	0.03	0.04	0.01	0.628
At the class level							

Bacilli	76.41	86.72	86.25	90.71	91.49	5.51	0.346
Clostridia	10.73	3.32	3.80	2.47	2.85	3.66	0.490
Gammaproteobacteria	2.03	2.57	5.45	2.05	1.89	1.94	0.666
Actinobacteria	5.16	0.99	1.16	1.67	1.21	1.74	0.418
Verrucomicrobiae	1.27	0.91	1.13	1.41	1.05	0.23	0.595
Betaproteobacteria	0.03	3.30	0.14	0.01	0.03	1.47	0.441
Bacteroidia	0.72	0.42	0.46	0.53	0.39	0.15	0.514
Alphaproteobacteria	0.93	0.35	0.39	0.36	0.08	0.32	0.474
Firmicutes unclassified	1.24	0.47	0.07	0.09	0.08	0.49	0.400
Negativicutes	0.16	0.39	0.37	0.24	0.44	0.15	0.640
Others	1.33	0.56	0.78	0.45	0.49	0.26	0.141
At the order level							
Lactobacillales	68.94	83.32	83.73	87.98	90.91	6.22	0.159
Staphylococcales	6.58	2.53	1.60	2.51	0.36	1.95	0.257
Burkholderiales	0.29	1.89	3.85	0.39	0.55	1.64	0.506
Enterobacterales	1.65	1.09	1.39	1.57	1.24	0.44	0.894
Oscillospirales	3.87	0.58	0.54	0.57	0.52	1.43	0.396
Corynebacteriales	3.77	0.28	0.22	1.27	0.44	1.39	0.360
Verrucomicrobiales	1.27	0.91	1.13	1.41	1.05	0.23	0.595
Clostridiales	2.04	1.11	1.15	0.39	0.44	0.69	0.461
Peptostreptococcales Tissierellales	0.53	0.41	1.35	0.62	0.97	0.43	0.546
Lachnospirales	1.09	0.72	0.53	0.66	0.66	0.18	0.272
Others	9.97	7.16	4.51	2.64	2.85	2.75	0.308
At the family level							
Lactobacillaceae	65.54	66.89	73.36	83.31	81.90	7.39	0.325
Streptococcaceae	1.37	7.89	6.17	1.04	4.32	1.79	0.056
Enterococcaceae	1.31	8.43	4.04	2.35	3.70	2.13	0.203
Staphylococcaceae	6.58	2.53	1.60	2.51	0.36	1.95	0.257
Enterobacteriaceae	1.47	1.02	1.31	1.40	1.22	0.39	0.936
Akkermansiaceae	1.27	0.91	1.13	1.41	1.05	0.23	0.592
Corynebacteriaceae	3.59	0.26	0.22	1.27	0.44	1.32	0.358
Lachnospiraceae	1.92	1.01	0.67	0.85	0.78	0.45	0.309
Burkholderiaceae	0.18	0.45	2.99	0.26	0.33	1.23	0.450
Ruminococcaceae	2.70	0.38	0.38	0.27	0.32	1.04	0.411
Others	14.07	10.22	8.15	5.34	5.59	3.95	0.514
At the genus level							
Ligilactobacillus	28.85	29.11	32.04	40.10	53.14	9.46	0.349
HT002	14.33	22.13	20.97	25.53	19.15	8.04	0.898
Lactobacillus	18.76	8.50	9.95	15.73	6.02	7.67	0.754
Streptococcus	1.34	7.72	6.15	1.03	4.28	1.79	0.060
Enterococcus	1.31	8.43	4.04	2.35	3.70	2.13	0.203
Limosilactobacillus	1.92^{ab}	4.50^{ab}	8.70^{a}	1.22^{b}	2.72^{ab}	1.67	0.034

Staphylococcus	6.49	2.53	1.56	2.43	0.33	1.93	0.257
Weissella	1.55	2.28	1.41	0.50	0.70	0.74	0.469
Akkermansia	1.27	0.91	1.13	1.41	1.05	0.23	0.592
Corynebacterium	3.59	0.26	0.22	1.27	0.44	1.32	0.358
Others	20.59	13.63	13.82	8.43	8.47	5.50	0.550

Note:"Others" includes phyla, classes, orders, families or genera beyond the top 10.

Supplement 3. Neurotransmitter and hormone levels in different tissues in the different groups (ng/mL)

**Item**	**Groups**					**SEM**	***P***
	**CON**	**PSE**	**PT**	**VR**	**VR+PT**		
Hypothalamus							
acetylcholine	90.51	104.75	78.73	77.72	75.35	8.42	0.114
serotonin	6.11	1.59	4.11	1.79	2.83	1.14	0.061
EN	3.85	2.90	4.89	3.23	2.39	0.64	0.099
NE	30.26^{ab}	21.62^{bc}	39.04^{a}	15.89^{c}	15.50^{c}	3.35	< 0.001
cortisol	8.90	16.57	12.77	9.06	8.26	2.92	0.250
Cerebellum							
acetylcholine	80.54	86.66	132.40	98.90	56.83	27.14	0.447
serotonin	70.00	40.33	59.36	47.44	63.44	12.78	0.488
EN	8.41	8.72	12.13	15.70	5.31	2.71	0.112
NE	35.90	30.17	37.03	19.80	26.83	5.14	0.155
cortisol	30.61	35.14	42.86	25.38	29.58	5.33	0.224
Kidney							
acetylcholine	109.38	113.86	184.21	274.32	83.35	47.61	0.064
serotonin	191.93^{ab}	116.31^{b}	131.97^{b}	160.53^{ab}	221.31^{a}	21.10	0.013
EN	12.31^{ab}	33.11^{a}	13.03^{ab}	10.91^{ab}	0.88^{b}	5.90	0.016
NE	496.48	302.04	485.11	309.14	290.38	84.23	0.229
cortisol	30.60	19.35	18.64	37.29	17.61	6.46	0.159
Intestinal Contents							
acetylcholine	29.69	14.30	27.87	17.87	24.40	6.53	0.426
serotonin	93.77^{b}	155.53^{ab}	116.63^{ab}	99.97^{b}	281.55^{a}	40.33	0.021
EN	3.93^{a}	3.49^{ab}	2.99^{ab}	1.49^{b}	1.95^{ab}	0.50	0.012
NE	29.12	7.10	10.37	6.30	21.31	11.96	0.603
cortisol	66.95	78.67	37.05	87.46	55.19	17.56	0.313
Serum							
acetylcholine	29.09	14.16	16.74	17.20	17.31	3.93	0.105
