# Supplementary material for: The impact of active components from Piper sarmentosum on the growth, intestinal barrier function, and immunity of broiler chickens
Source: Anim Biosci. 2025 Feb 27;38(7):1522–34. doi: 10.5713/ab.24.0736 (PMC12229918; doi:10.5713/ab.24.0736)
Supplement: Supplementary file 3 [file ab-24-0736-Supplementary-3.pdf]

Staphylococcus	6.49	2.53	1.56	2.43	0.33	1.93	0.257
Weissella	1.55	2.28	1.41	0.50	0.70	0.74	0.469
Akkermansia	1.27	0.91	1.13	1.41	1.05	0.23	0.592
Corynebacterium	3.59	0.26	0.22	1.27	0.44	1.32	0.358
Others	20.59	13.63	13.82	8.43	8.47	5.50	0.550

Note:“Others” includes phyla, classes, orders, families or genera beyond the top 10.

Supplement 3. Neurotransmitter and hormone levels in different tissues in the different groups (ng/mL)

**Item**	**Groups**					**SEM**	***P***
	**CON**	**PSE**	**PT**	**VR**	**VR+PT**		
Hypothalamus							
acetylcholine	90.51	104.75	78.73	77.72	75.35	8.42	0.114
serotonin	6.11	1.59	4.11	1.79	2.83	1.14	0.061
EN	3.85	2.90	4.89	3.23	2.39	0.64	0.099
NE	30.26^{ab}	21.62^{bc}	39.04^{a}	15.89^{c}	15.50^{c}	3.35	< 0.001
cortisol	8.90	16.57	12.77	9.06	8.26	2.92	0.250
Cerebellum							
acetylcholine	80.54	86.66	132.40	98.90	56.83	27.14	0.447
serotonin	70.00	40.33	59.36	47.44	63.44	12.78	0.488
EN	8.41	8.72	12.13	15.70	5.31	2.71	0.112
NE	35.90	30.17	37.03	19.80	26.83	5.14	0.155
cortisol	30.61	35.14	42.86	25.38	29.58	5.33	0.224
Kidney							
acetylcholine	109.38	113.86	184.21	274.32	83.35	47.61	0.064
serotonin	191.93^{ab}	116.31^{b}	131.97^{b}	160.53^{ab}	221.31^{a}	21.10	0.013
EN	12.31^{ab}	33.11^{a}	13.03^{ab}	10.91^{ab}	0.88^{b}	5.90	0.016
NE	496.48	302.04	485.11	309.14	290.38	84.23	0.229
cortisol	30.60	19.35	18.64	37.29	17.61	6.46	0.159
Intestinal Contents							
acetylcholine	29.69	14.30	27.87	17.87	24.40	6.53	0.426
serotonin	93.77^{b}	155.53^{ab}	116.63^{ab}	99.97^{b}	281.55^{a}	40.33	0.021
EN	3.93^{a}	3.49^{ab}	2.99^{ab}	1.49^{b}	1.95^{ab}	0.50	0.012
NE	29.12	7.10	10.37	6.30	21.31	11.96	0.603
cortisol	66.95	78.67	37.05	87.46	55.19	17.56	0.313
Serum							
acetylcholine	29.09	14.16	16.74	17.20	17.31	3.93	0.105

serotonin	5196.00	3966.30	3909.50	3898.50	5458.70	1301.89	0.838
EN	34.62[a]	5.89[b]	6.60[b]	28.93[ab]	13.58[ab]	5.78	0.005
NE	70.77	80.94	91.44	51.73	45.41	21.20	0.520
cortisol	5.07	7.59	6.06	6.03	6.28	1.00	0.532

Abbreviations: NE, norepinephrine; and EN, epinephrine.
