# Supplementary material for: The impact of active components from Piper sarmentosum on the growth, intestinal barrier function, and immunity of broiler chickens
Source: Anim Biosci. 2025 Feb 27;38(7):1522–34. doi: 10.5713/ab.24.0736 (PMC12229918; doi:10.5713/ab.24.0736)
Supplement: Supplementary file 4 [file ab-24-0736-Supplementary-4.pdf]

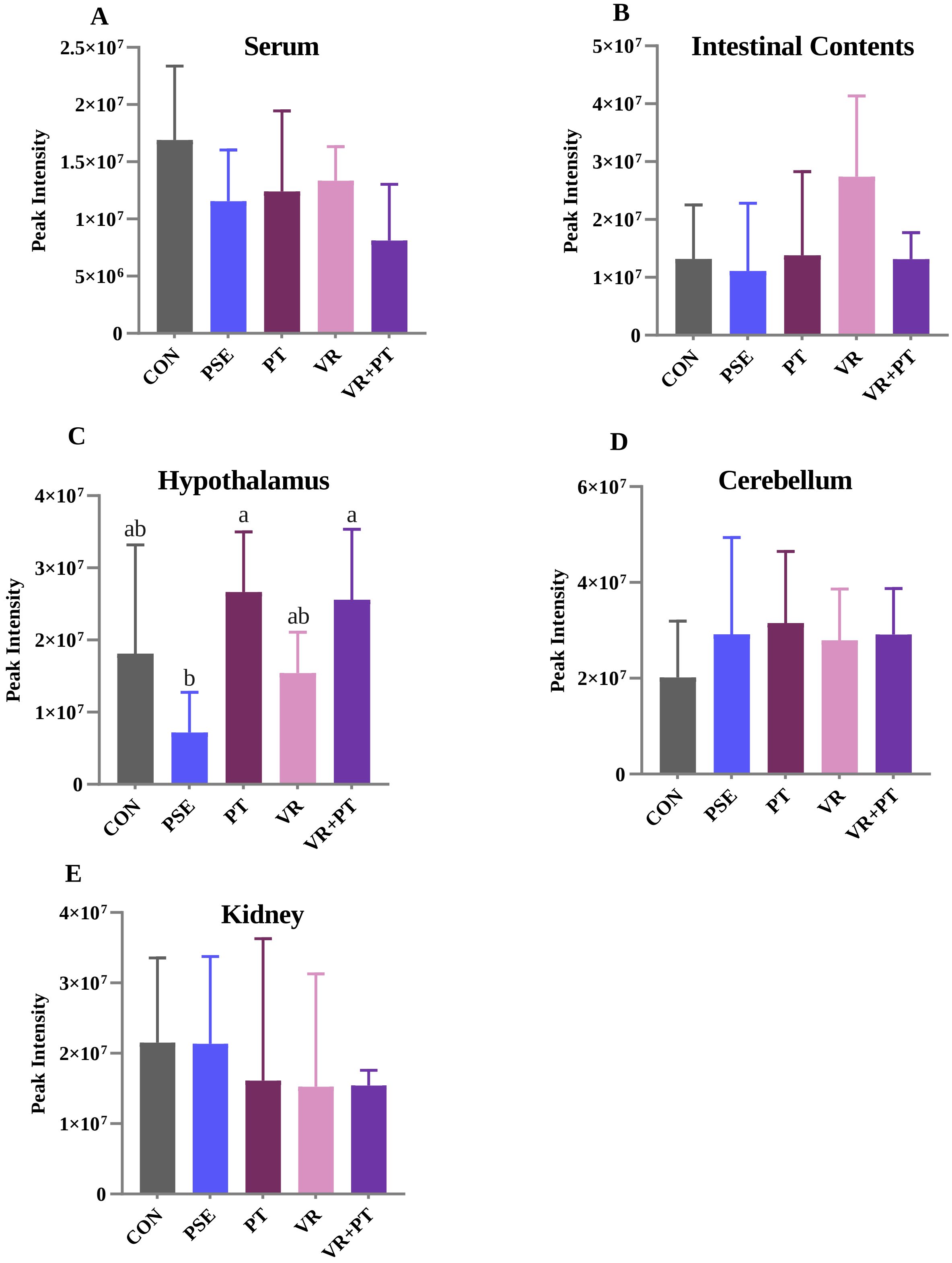

Supplement 4. Relative ACTH level in different parts or tissues of chickens in the different groups as quantified by UPLC-QQQ-MS. (A) Relative ACTH level in serum; (B) Relative ACTH level in intestinal contents; (C) Relative ACTH level in hypothalamus; (D) Relative ACTH level in cerebellum; (E) Relative ACTH level in kidney. Data are presented as mean ± SD. Different letters within the figure indicate significant differences (ANOVA; $P < 0.05$; $n = 5$).
